# Active Haptic Perception in Robots: A Review

**DOI:** 10.3389/fnbot.2019.00053

**Published:** 2019-07-17

**Authors:** Lucia Seminara, Paolo Gastaldo, Simon J. Watt, Kenneth F. Valyear, Fernando Zuher, Fulvio Mastrogiovanni

**Affiliations:** ^1^Department of Electrical, Electronics and Telecommunication Engineering and Naval Architecture, University of Genoa, Genoa, Italy; ^2^School of Psychology, Bangor University, Bangor, United Kingdom; ^3^Department of Computer Science, Institute of Mathematics and Computer Science, University of São Paulo, São Carlos, Brazil; ^4^Department of Computer Science, Bioengineering, Robotics, and Systems Engineering, University of Genoa, Genoa, Italy

**Keywords:** haptic perception, active touch, exploration, robot touch, humans and robots, sensorimotor control

## Abstract

In the past few years a new scenario for robot-based applications has emerged. Service and mobile robots have opened new market niches. Also, new frameworks for shop-floor robot applications have been developed. In all these contexts, robots are requested to perform tasks within open-ended conditions, possibly dynamically varying. These new requirements ask also for a change of paradigm in the design of robots: on-line and safe feedback motion control becomes the core of modern robot systems. Future robots will learn autonomously, interact safely and possess qualities like self-maintenance. Attaining these features would have been relatively easy if a complete model of the environment was available, and if the robot actuators could execute motion commands perfectly relative to this model. Unfortunately, a complete world model is not available and robots have to plan and execute the tasks in the presence of environmental uncertainties which makes sensing an important component of new generation robots. For this reason, today's new generation robots are equipped with more and more sensing components, and consequently they are ready to actively deal with the high complexity of the real world. Complex sensorimotor tasks such as exploration require coordination between the motor system and the sensory feedback. For robot control purposes, sensory feedback should be adequately organized in terms of relevant features and the associated data representation. In this paper, we propose an overall functional picture linking sensing to action in closed-loop sensorimotor control of robots for touch (hands, fingers). Basic qualities of haptic perception in humans inspire the models and categories comprising the proposed classification. The objective is to provide a reasoned, principled perspective on the connections between different taxonomies used in the Robotics and human haptic literature. The specific case of active exploration is chosen to ground interesting use cases. Two reasons motivate this choice. First, in the literature on haptics, exploration has been treated only to a limited extent compared to grasping and manipulation. Second, exploration involves specific robot behaviors that exploit distributed and heterogeneous sensory data.

## 1. Introduction

There is a compelling case for using principles of human haptic perception—active touch—to inspire the development of robot haptic systems. Human haptic exploration is efficient, robust to noise, yet adapts rapidly to changing conditions (Prescott et al., 2011). Moreover, human hand control is the archetypal general-purpose sensorimotor system. It uses biological mechanisms to achieve a remarkable degree of functional flexibility, across huge variations in conditions, that remains elusive in “intelligent machines.” Key to this capability (over-and-above the hand's anatomy) are the neural control processes underlying hand function. It is valuable therefore to consider the approaches taken to robot haptics, and the main challenges, in light of these “organizational principles” of human hand control. In this paper, we analyse Robotics-related and human haptic literature to discuss about how human-centered studies can inform the design of novel robot behaviors when haptic processes are needed.

Haptics provides useful information under a wider set of circumstances than is sometimes appreciated (Lederman and Klatzky, 2009). First, and most obviously, haptics can be the only available signal, such as in poorly lit environments, or when reaching into a bag. Second, even when other senses are available, haptics provides direct information about properties of objects that are poorly (or at least very indirectly) specified by other senses. This is because haptics, uniquely among human senses, involves physical interaction with objects, allowing properties such as friction and compliance to be sensed. Third, haptics contributes to sensorimotor processing even when it provides redundant information to other senses. Our perception of the size of an object held in our hand, for example, is derived by integrating information from vision and haptics, in a statistically optimal fashion, resulting in the most precise estimate possible in a given situation (Ernst and Banks, 2002). Thus, just as ventriloquism reveals that people routinely integrate visual and auditory signals *even when other senses are available* (Alais and Burr, 2004), we constantly make use of haptic information to optimize perception (though we may not be aware of it).

A primary property of human haptic perception is that it is active: sensory signals are acquired through purposive movements, made to actively explore the world (Prescott et al., 2011; Bajcsy et al., 2018). This places haptics at the nexus of perceiving and acting—two facets that are sometimes explicitly separated in human neuroscience (Trevarthen, 1968; Marr and Nishihara, 1978; Bridgeman et al., 1981; Goodale et al., 1991; Milner, 1995; Jeannerod, 1997). The need for highly organized and purposeful movements for haptic object recognition is evident in findings from human neuropsychology. Binkofski et al. (2001) demonstrate that impaired haptic object recognition following damage to specific parts of parietal cortex is associated with atypical exploratory movement strategies. Patients showed decreased frequency and regularity of digit movements, and increased “exploration space,” consistent with an inability to identify and exploit areas of “object space” that are most diagnostic for object recognition. Critically, impaired explorative strategies were not attributable to low-level sensory or movement deficits, but reflected the loss of ability to organize movements as an effective information-seeking plan. Thus, as Prescott et al. (2011) note, understanding haptics requires understanding a system in which there is “no strong demarcation between sensation and action.”

The active nature of haptics presents key challenges to haptic-sensing robots. It is necessary to determine not only what the properties of the sensors should be, but also what information is required in a given situation, and which actions should be performed to elicit it. Gibson (1950) described active perception in the context of vision, noting that optic flow signals to observer motion and the structure of the environment are acquired by an observer who moves, and therefore directly influences the acquired information. More generally, active perception involves not only active control of the sensory apparatus, but also dynamic reconfiguring of the internal parameters of the system to suit the current task (Coates et al., 2008; Prescott et al., 2011; Bajcsy et al., 2018). Anyone who has searched their child's Lego™ tray for *that* piece, for example, will appreciate the benefit conferred by knowing what to look for!

The relative difficulty of this wider challenge of goal-directed perception seems related to the paucity of general-purpose intelligent machines. Machine vision algorithms, for example, can match or sometimes exceed human performance on relatively constrained tasks, such as image classification. However, for more open-ended tasks, where the agent must decide what information to seek and which actions to perform, progress is comparatively limited—see Bajcsy et al. (2018)'s discussion of a machine-vision guided system for making a salad! In haptic sensing, there are no signals without movements, and so the problem of seeking information is inherent and cannot be avoided in robot haptics. Systems will be needed that perform functions analogous to maintaining goals, attending selectively to information, decision-making, etc.

Humans use specific movement strategies to extract information about object properties during haptic exploration. These stereotyped movements, which Lederman and Klatzky (1990) referred to as exploratory procedures (EPs), highlight several challenges in interpreting haptic signals. Examples of EPs include prodding an object with the fingertips along its surface-normal, to elicit information about compliance or hardness, and making side-to-side movements across an object, to recover surface texture. A key “problem” in both of these examples is that the incoming signals reflect the simultaneous activity of multiple sensory systems. Extracting meaningful information (building up an internal representation of an object, for example) requires combining information from different sensors. This presents a version of Bishop Berkeley's sensory correspondence problem, where the relationship between fundamentally different sensory signals must be known in order to treat those signals as relating to a single, common object or property (Berkeley, 1709; Ernst, 2007).

Relatedly, raw sensor outputs do not normally map directly onto meaningful “psychological variables” that relate to our functional understanding of the world. Consider the task of estimating the size of an object held in the hand. This information is sensed by changes in muscle length, finger-joint angles etc., but it does not make sense for the brain to have conscious access to these raw signals. Rather, perception requires access to higher-order properties relevant to the task (here, size). Similar arguments can be made for other surface properties. The raw output of individual or groups of mechanoreceptors is not the desired output. Rather, we wish to know the properties of surfaces in meaningful terms such as texture and compliance (Johansson and Pruszynski, 2014). This is somewhat analogous to the well-known size-constancy problem in human vision: we need to perceive the size of objects in the world, not their size on the retina (which depends on distance). Indeed, extraction of “higher-level” properties, may underly human effectiveness at tasks such as tool use, allowing haptic perception to operate at the level of object properties, independent of the end effector with which they are felt (Arbib et al., 2009; Takahashi and Watt, 2017).

Ultimately, assuming sensory information is used to build useful internal representations of objects, information must be transformed into units that can be combined with other sensory signals (e.g., from vision) to create multimodal representations in higher-order, psychologically meaningful units (Landy et al., 1995). Evidence from human neuroimaging suggests that haptic and visual object recognition involve both distinct and overlapping brain mechanisms (Amedi et al., 2002; James et al., 2002; Hernandez-Perez et al., 2017).

Integrating information across multiple sensors and sensory systems also provides a reduction in the dimensionality of the information, without which the computational challenge of interpreting sensory signals would be intractable (Wolpert and Ghahramani, 2000). For example, Prescott et al. (2011) describe work by Kappers (2011) showing that surface curvature is judged on the basis of a reduced “dataset” from what is available in principle at the sensors. They note that such dimension reduction has been proposed by Hayward (2011) as reflecting a general principle, whereby the brain makes “simplifying assumptions” that are necessary to manage the complexity of the totality of information that can potentially be acquired by the haptic sensory system (what Hayward calls the “plenhaptic function”; analogous to the plenoptic function, or light field, in optics). In other words, information is discarded at the level of the sensors, and in neural processing beyond, to render the problem tractable.

Inherent in the above discussion, haptic perception extends beyond extracting meaning from isolated estimates of object properties to include building up a “holistic picture” that supports activities such as object recognition, manipulation and so on. This poses questions about the frame of reference in which information is encoded. Haptics again differs from vision in this regard in that such representations must be built from samples that may be discrete in terms of the properties they specify, and their spatial locations (and may come from different hands). This implies that tasks such as object recognition are best served by a stable (i.e., slow changing), object- or world-based frame of reference/coordinate system, which maintains unchanging properties of objects over movements of the hand (the sensor). At the same time, exploratory hand movements need to reflect moment-by-moment positions of the digits with respect to the object, which might be better suited to an egocentric frame of reference, updated rapidly. Evidence suggests that the systems-level functional organization of the brain is consistent with this somewhat task-dependent solution. Object recognition and the control of actions (and information about where things are), respectively, largely rely on anatomically and functionally separable brain systems (Trevarthen, 1968; Ungerleider and Mishkin, 1982; Goodale et al., 1991; Milner, 1995). This organization duplicates some information, but in distinct forms that are better suited for given tasks, allowing, for example, for rapid and accurate motor responses where necessary.

The requirement for dimension reduction also exists for controlling motor output. At the conscious level, it is intractable to plan the trajectory of a grasping movement, for example, in terms of individual joint angles and torques. Instead, it is thought that we plan movements in the much lower dimensional space of our end effectors (here, our hand and fingers), while unconscious processes are responsible for the specific transformations required to achieve this. Indeed, relatively recent evidence from electrophysiological studies in primates suggests that movements of end effectors may even be represented in primary motor cortex (M1), previously thought to reflect the “commands” sent to the muscles (Umiltà et al., 2008). Relatedly, the relationships between the firing patterns of specific cells in M1 and electrical activity in the muscles of the arm/hand have been shown to exhibit task-specificity, consistent with the idea that goals and context can change the ultimate effect of outgoing signals from M1 on muscle contractions (Quallo et al., 2012). Of course, at some level individual muscles must be controlled, and so there is a dimension-reduction problem here too. The related ideas of motor primitives, and motor synergies—characteristic spatiotemporal patterns of “control signals,” potentially at multiple levels (activation of nearby muscles, up to higher-level co-ordination), which can be combined to build complex movements—provide plausible ways in which this problem might be solved (Thoroughman and Shadmehr, 2000; Wolpert and Ghahramani, 2000; Santello et al., 2016).

A similar complexity problem to controlling movements exists at the level of *selecting* which movement to make, from the potentially infinite number of possibilities. Recently, it has been proposed that these processes—movement specification and selection—may be carried out using common brain mechanisms. Numerous data indicate that the same neural populations responsible for specifying the spatiotemporal parameters of forthcoming actions causally contribute to action selection (Cisek and Kalaska, 2005; Hanks et al., 2006; Scherberger and Andersen, 2007; Pesaran et al., 2008; Pastor-Bernier and Cisek, 2011; Thura and Cisek, 2014; Christopoulos V. N. et al., 2015). These data motivate the hypothesis that decisions about which actions to perform are made by resolving competition between concurrently active sensorimotor representations that specify how actions can be performed (Cisek, 2007; Cisek and Kalaska, 2010; Christopoulos V. et al., 2015). From a biological perspective, this idea has several attractive features, including making dual use of the same neural resources, and unifying the problems of action planning (how to move) and selection (which actions to perform). Such insights might be useful for Robotics. An artificial system that converts sensory inputs into motor parameters capable of being used to control future actions could also be programmed to make action choices on the basis of those same units. Of course, to make adaptive, goal-directed “choices,” the problem of how to implement high-level context signals (i.e., goals) remains.

As noted earlier, motor control and sensory input are interlinked in human haptic sensing: purposive movements provide information to build internal representations of properties of the world and guide future movements, in a closely coupled manner. It is likely to be valuable, then, to consider general emergent principles across these “domains.” Dimension reduction is one such. Indeed, to the degree that these lower-dimensional spaces for motor control and perception are common, they offer a computational framework for considering perception in terms of the opportunities for actions that objects provide, similar to Gibson's long-standing idea of “affordances” (Gibson, 1950). Moreover, an important common principle of human motor control and perception that has emerged in the last 25 years is that both are well characterized as optimization problems (Knill and Richards, 1996; Harris and Wolpert, 1998; Todorov and Jordan, 2002). The motivating assumption is that neural systems are corrupted by noise/uncertainty, at all levels (knowledge of the world, sensory signals, motor outputs, and any intervening neural processes such as coordinate transformations). Thus, the “job” of planning movements, or recovering properties of the world, is rendered probabilistic in nature, and can be thought of as decision-making under uncertainty (Trommershäuser et al., 2008; Wolpert and Landy, 2012). Bayesian inference provides a principled framework for optimally solving such problems, and there is now considerable empirical evidence that the human brain takes noise into account appropriately, combining sensory signals, and programming movements, in ways that resemble Bayes-optimal solutions, see Ernst and Bülthoff (2004), Wolpert and Landy (2012), and Wolpert and Ghahramani (2000). In particular, Wolpert and Ghahramani (2000) describe an optimal control framework for motor control, which may provide a unifying framework for human haptics. They consider the problem of planning a movement (based on sensory input) and carrying it out using sensory feedback about the state of the moving limb (in low-dimensionality units), which is inherently subject to sensory processing delays. Their work highlights the importance of forward models, in particular, generated from a copy of the motor commands, and used to predict the future outcome of the movement (Wolpert et al., 1995). These forward models result in error signals between actual and expected outcomes, which can support calibration, and learning. Moreover, the predicted state of the limb can be combined with sensory feedback to estimate its state optimally, given uncertainty in all estimates, and despite delayed sensory feedback.

## 2. Closed-Loop Sensorimotor Control of Robot Hands: A New Taxonomy

The Introduction gave us an overview of the fundamental conceptual organizational principles of human haptic perception, which is intrinsically connected with the capability of attending selectively to information, making decisions, planning, producing movements and executing tasks in the presence of environmental uncertainties. All of this requires coordination between motor and sensory-feedback systems.

**Figure 1:**
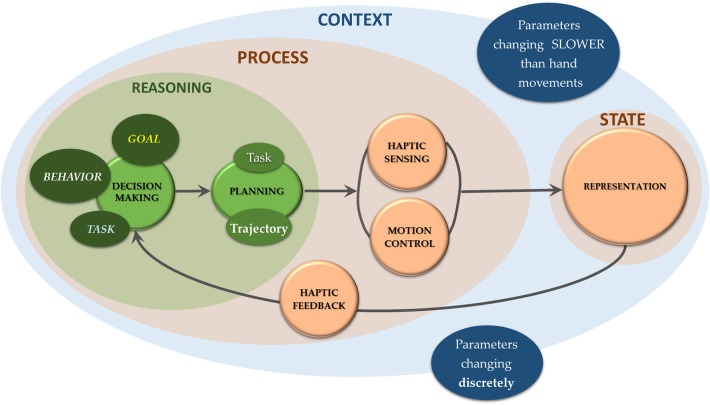
The closed-loop sensorimotor control of robots for touch is intended as a sequence of system *states*, represented through features. The *process* step includes all dynamical processes in-between two states and it is enabled by *haptic feedback*. This step includes a *reasoning* stage in which choices are made on how to move (*decision making* and *planning*), and a controlled *haptic sensing*-while-*moving* stage to acquire sensor data to be processed for system representation. Motion commands are tailored to prevailing movement *contexts*, defined by discrete or slowly changing parameters with respect to the movements for touch (Wolpert and Ghahramani, 2000).

While debates on human sensorimotor control are still open, in the case of robots the haptic processing workflow must nonetheless be clearly defined, because robots are guided by algorithms. For a roboticist, it is natural to think of closed-loop sensorimotor robot control as comprising the following pipeline: sensing, processing, state representation, reasoning and acting. In a robot, perceptual data are organized into data structures. Extracted features are represented as part of the system state and enable checking that control objectives are met. We thus describe the sensorimotor robot control loop at a high level as a sequence of “states,” where the transition between two subsequent states is governed by a “process” step, which therefore modifies the state itself. In line with what was posited by Wolpert and Ghahramani (2000), the state directs the next motion step and motion commands depend on the context and on the chosen “behavior,” “goal” and the corresponding “tasks” (Figure 1).

### 2.1. The State: The Concept of Representation

In the proposed conceptualization, the system state is in-between two steps of motion of the robot for touch. Depending on the context-behavior-goal-task, both rough sensory data and specific features extracted from sensor measurements during hand motion help in defining the overall (haptic) state. The state therefore enables checking whether control objectives are met, and this output defines what information is fed back to the reasoning stage that guides new actions. Consistent with theoretical accounts of human sensory-feedback systems where lower-dimensional representations are derived from purposeful exploratory movements (Wolpert and Ghahramani, 2000), the robot-related system state directly connects with the concept of an internal haptic representation. Like in humans, for robots the haptic representation is task-dependent and might include spatial properties (i.e., poses of both touched object and the hand), and/or canonical properties of the touched object (mainly geometry and material of the object itself). The state, in so far as it includes haptic information, is represented accordingly. Focusing on the touched object, its haptic representation is based on a number of haptically-specified features that are constituents of the “object proper” (whole object), which itself has spatial properties, including location and orientation about a principal axis.

**Figure 2:**
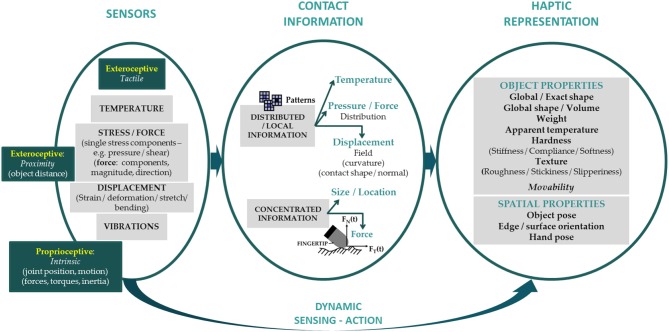
The artificial sensory apparatus related to robot touch dynamically measures information about contact: the picture illustrates the route from sensor outputs to a system haptic representation. The sensor block includes an improved taxonomy, borrowing some details from a scheme introduced by Kappassov et al. (2015) (with permission from the authors). It contains information about all sensor types that are relevant for haptics (i.e., both proprioceptive and exteroceptive sensors) and specifies what tactile sensors in particular measure at their positions (raw sensor data), i.e., temperature, stress/force, displacement and/or vibrations. Distributed or concentrated information related to contact is generally the result of applying algorithms to sensor outputs. Higher level feature extraction allows for task-dependent haptic representation of the system state, including haptically accessible *object properties* and the *spatial properties* of both the touched object and the agent for touch. All categories for object properties (in bold) are derived from Lederman and Klatzky (2009). The intent here is to present an overall perspective, opening different routes on possible integrated approaches. Analyzing how to practically integrate these approaches with their benefits and drawbacks is beyond the scope of this paper.

The concept of haptic representation is directly connected with the concept of haptic measurement (Figure 2). Starting with touch, high resolution and distributed tactile information mimicking human sense of touch requires the use of coordinated groups of tactile sensors (typically, in the form of arrays). The literature on tactile sensing is extensive, and recent advances on tactile sensors for Robotics have been discussed elsewhere (Dahiya et al., 2010; Yousef et al., 2011; Kappassov et al., 2015; Martinez-Hernandez, 2016).

Classification of sensory outputs is of particular relevance for the scope of this paper. Contributions from other, non-tactile sensors are considered when they are inextricably bound with haptic processing and representation. Consistent with a biomimetic perspective, we include proximity sensors mimicking whiskers and antennae (from animal active touch) in the category of exteroceptive sensors, and add proprioceptive sensors. Even if intrinsic sensors can give approximate information about interaction forces, extrinsic tactile sensors give much more precise information about interaction properties (Wettels et al., 2014).

It is noteworthy that sensory information is rarely used in its raw form: algorithms are used to extract features from sensory data. This is akin to the data-reduction processes that are thought to characterize human sensory-feedback control systems, as discussed above. At the lowest level, tactile sensor arrays can be used to find contact locations with a high resolution, and to track variation of contact points. Model-based approaches can be used to retrieve higher-level information related to contact features, such as contact force distributions (Seminara et al., 2015) or contact shape (Khan et al., 2016; Wasko et al., 2019). In all cases, such algorithms are needed to embed 3D sensor locations into a lower dimensional space representing the robot surface, and a further processing step is required to move toward a higher dimensional space by computing features. General techniques of data pre-processing can be used, such as scaling, dimensionality reduction, spatial filtering and/or thresholding. Alternatively, pattern recognition algorithms have been used extensively for different purposes, including object features. Finally, features can be combined through additional processing steps, e.g., concatenation, voting and joint probability. A comprehensive overview of the different approaches used to extract high-level information from haptic sensory data in Robotics is beyond the scope of this review, and has been discussed elsewhere (Hoelscher et al., 2015; Kappassov et al., 2015; Luo et al., 2017). Haptically accessible object properties and system spatial properties define the system state, i.e., the representation, which—as for humans—contains all the relevant time-varying information needed for control, through sensory feedback, and predict the future state of the system (Wolpert and Ghahramani, 2000).

To define categories for the canonical properties of a touched object, research on human haptics is informative (Lederman and Klatzky, 2009): haptically specified object properties include “geometric” (e.g., object size and shape), “hybrid” (e.g., weight), and “material” (e.g., surface, mechanical, thermal) properties (see Figure 2). Regarding geometric properties, the size and shape of objects can be considered on two scales: objects smaller than the fingertip, thus revealing their shape by skin indentation, and objects with contours that extend beyond this scale, for which shape perception reflects the contribution of proprioceptive inputs. Out of the various geometric properties, curvature has received the most attention. Size can be measured using a number of metrics, namely total area, volume, or perimeter. Shape is particularly hard to characterize and the orientation of certain object features, like its edges, vertices and contours, is relevant for shape recognition. As for material properties, among the different ways of characterizing surface textures, roughness has been investigated the most. The roughness percept contains information about the properties of the touched surface in relation to how the object is manually explored. The mechanical compliance of a touched object refers to its deformability under force, and measuring differences in thermal properties is necessary for material discrimination. Finally, the perceived weight of an object indicates its density and structure. *Movability* has been added to account for an interesting salient feature from the human haptic literature. In particular, it has been found that if an object moves upon touch, this greatly adds to the perception of indeed touching an object and not just the background (Pawluk et al., 2011).

Task-dependent system representations might include the spatial properties of both the touched object and the agent for touch. In general, those properties are mainly related to position and orientation (i.e., the “pose”) of both the agent for touch and the object itself (or some of its parts, such as edges and surfaces). These features are important to represent for the control of specific robot behaviors such as grasping, and certain exploratory procedures.

### 2.2. The Process: All Around Changing the State

**Figure 3:**
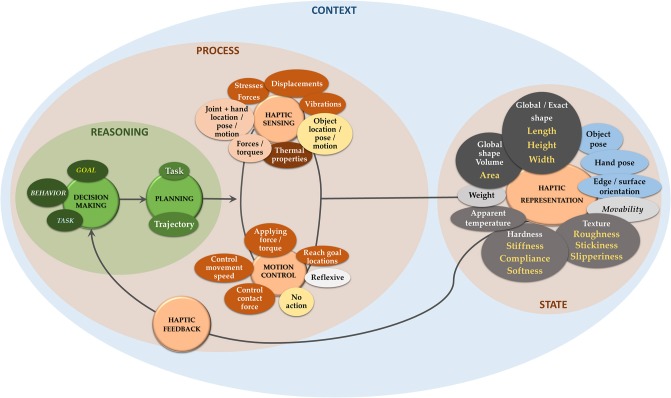
Overview of the proposed taxonomy. The *state* includes all categories described in section 2.1. Gray blocks among haptic features are related to the object: darkest are geometric, lighter is hybrid and mid gray blocks are related to material properties. For all gray blocks, top features belong to the taxonomy from Lederman and Klatzky (2009), at the bottom (yellow) are same category features as commonly found in the Robotics literature. Movability is added among gray blocks. Light blue blocks relate to spatial information. The *process* step includes both a first phase of choice, planning and triggering motion (corresponding to action planning and selection in humans), and a dynamic phase including all adjustments during sensorimotor control driven by sensory feedback. Blocks belonging to *Haptic Sensing* reproduce information given by all sensor categories of Figure 2: exteroceptive-mechanical (orange), exteroceptive-thermal (brown), exteroceptive-proximity (yellow), proprioceptive-dynamics/kinematics (pink). *Motion Control* first guides motion unfolding to reach goal locations in space. Reactive (orange) and reflexive (white) actions have been included in the motion control scheme for the sake of completeness. “No action” includes receptive mode through whiskers and antennae.

Recalling that our aim is to provide the reader with an overall functional picture of sensorimotor control of agents for touch (i.e., hands or fingers), Figure 3 illustrates the complete proposed dynamic framework. It is important to emphasize that the taxonomy defined in the figure is not a rigid scheme: it is rather an instance of the current state of the literature, which will develop with time and continued advancements. The purpose of this taxonomy is to generate an overall framework to be applied to specific examples of active exploration (section 3).

In the scheme of Figure 1 all aspects related to decision making (i.e., choice of behavior, high-level goals, and tasks) and motion planning (i.e., selection of the movement sequence within the space of possible solutions) are included in a “reasoning” stage^1^.

**Figure 4:**
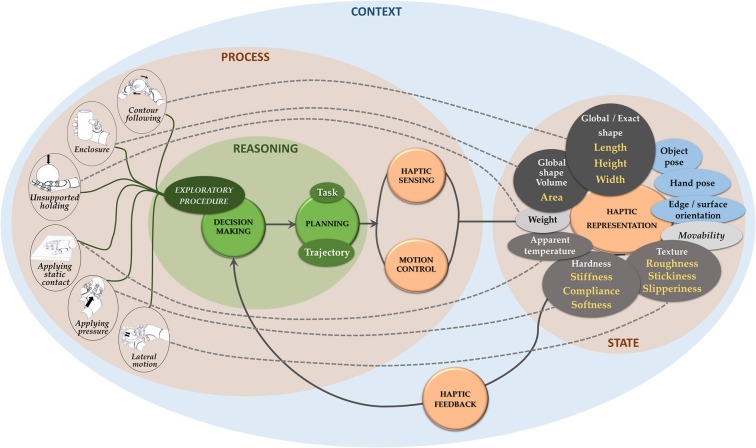
Exploratory procedures associated with haptic features as in Lederman and Klatzky (2009). Pictures on exploratory procedures have been borrowed from Lederman and Klatzky (1987) (with permission from the authors), where the human hand has been replaced with a robotic one.

When an object is in contact, an incessant coupling between heterogeneous, distributed sensing and robot behavior is needed to allow data processing and robot control units to apply appropriate interleaved sensing and action policies. Inspiration can be gained here from the science of human sensory systems, for example, from our understanding of how haptic exploratory movements are organized to extract specific object properties (Figure 4). Together with exploratory procedures, dynamic touch (e.g., grasping or wielding) can also be conceptualized within this scope (Turvey and Carello, 1995).

Therefore, in robots, action induced by motor control includes generating the desired motion of the end-effector during the contact event (controlling movement speed or contact force) and applying the desired forces/torques to accomplish a certain task. A variety of tasks can be carried out, from generating or following specific trajectories (e.g., contours) to exploring, manipulating, or grasping objects. In the case of active movements there are mainly two aspects of motor control which need to be included (Denei et al., 2015): maintaining contact with the body and planning the contact trajectory. It is noteworthy that controlling the contact force and the movement speed within the contact regime can be a possible solution to reduce the haptic rebound effects. Obviously for scenarios of physical human-robot interaction, such a phenomenon is also due to the peculiar choice of control algorithm.

Elements of a task-specific representation (section 2.1) are used as an input to the control algorithms used to accomplish a certain motor task. Robots need algorithms that can conveniently map the information provided by high-density, distributed heterogeneous sensing into a specific coding system (i.e., haptic feedback) oriented toward motion control. Details and models behind this dynamic process strongly depend on whether we deal with passive or active perception. In preliminary (passive) attempts to include information from artificial tactile sensory feedback to guide motion control of robot hands, motion control was oriented to a specific predefined state and sensory feedback was used only to allow for error compensation between the real and target states. Conversely, active robot sensorimotor control is closer to what humans do when purposefully moving their hands and fingers to enhance the perceptual characteristics of what they are touching (Lederman and Klatzky, 2009). In this case, sensor positions in space can be dynamically adjusted with local small repositioning movements to reduce perceptual uncertainty. This can be followed by explorative movements based on the outcomes of each perceptual decision-making step (Martinez-Hernandez et al., 2017). Bayesian approaches are mainly used for that, as discussed in the next section.

## 3. Active Exploration: Use Cases

In a typical Robotics-based design process, the scope is related to the optimization of a specific function of the artificial agent for touch. Therefore, the design is mostly governed by such principles as those related to some metric of optimality. In principle, the control of robot agents for touch does not necessarily employ biomimetic-related design concepts. Traditionally, motion control of robot hands or fingers makes use of concepts like synergies and dimensionality reduction only to a limited extent, and always to trade-off between the complexity in mechanical and control design and function. Optimizing certain haptics-based tasks or behaviors might lead to specialized mechanical structures for the robot device, whose appearance might differ quite a lot from that of the corresponding human agent. However, human-inspired principles might be useful when such behaviors as active exploration are under study, possibly related to contexts in which robots are requested to perform tasks within open-ended conditions and should be capable of interacting with objects, other robots, or humans.

Conversely, the constrained mechanical structure of the agent for touch has implications on the kind of control principles that can be implemented, thus influencing behaviors the agent can perform or the way in which specific tasks can be carried out. Anthropomorphic approaches become relevant when a task-driven approach is not possible as the task is not well-defined, or cannot be characterized in terms of estimated input models. Instead of solving a specific task in the most efficient way, the goal in this case is to provide a general purpose machine, suitable to manage a variety of possible behaviors and tasks in different contexts. This approach is certainly relevant for applications including humans into the sensorimotor control loop, for example when designing artificial prosthetic systems for the reconstruction of the sense of touch. The key aspect in this case is the set-up of a human-like artificial prosthesis for touch allowing its user to move and/or feel in a *recognizable* and *intuitive* way, which not only relates to hand functions but also to the concept of “embodiment.”

The following discussion is structured into two main categories: approaches conceived to optimize a specific haptic task with a specific platform for touch (“task-based”), and approaches based on anthropomorphic platforms for open-ended haptic behaviors (“structure-based”). The focus will be on such behaviors as active exploration, in that they inherently link to the active nature of human touch and the related principles of human motor control, relevant for the framework of this paper. Common to the two approaches, what enables the ability of an artificial agent for touch to perform a variety of tasks and behaviors is also the qualities of its sensing system. It is useful to start this discussion focusing on the required perceptual abilities of an artificial finger suitable for active touch, already observing from the perspective of a closed-loop approach.

### 3.1. Perceptual Abilities of the Artificial Agent for Active Touch

A broad overview of materials and sensing technologies for advanced biomimetic tactile sensing for prosthetic hands is reported by Chortos et al. (2016) and Li et al. (2017). In particular, Chortos et al. (2016) present a complete overview of current research on materials and devices designed for mimicking the skin's ability to sense and generate signals. A fully biomimetic approach is used, starting from sensors (illustrating biomimetic strategies to improve the performance of the sensing device and provide skin-like functionality), moving to the electronics for reading sensory data and encoding biomimetic output, and ending up with revising all current methods for interfacing the artificial sensing system with the human nervous system.

When it comes to active control in Robotics, a human is not included in the sensorimotor control loop. However, haptic perception in humans might inspire the design of sensing systems suitable for active touch. Leaving classical modular compartments (e.g., sensations, percepts, and cognitions), the proposed functional distinction between *what* and *where* systems proposed by Lederman and Klatzky (2009) is useful for the framework presented in this paper and already inspired the building blocks of the haptic representation of the touched surface or object (section 2.1). The spatial and temporal resolving capacities of the skin are relevant for the haptic perception of object and surface properties. The precision with which humans can localize features and contacts is affected by the spatial resolving capacity of the human skin: the spatial acuity for human hands is around 1–2 mm for the fingertips and about 1 cm for the palm (Lederman and Klatzky, 2009). About the temporal resolving capacity of the skin, a common measure indicates that people can resolve successive taps on the skin at a frequency lower than 200 Hz. High resolution distributed tactile sensing might enable human-like perception: to make an example, as roughness perception in humans is determined by the spatial distribution of the textural elements rather than by temporal factors (Lederman and Taylor, 1972), appreciating spatial information distribution through an artificial skin may be needed. Certainly, adequate spatial resolution is necessary for perception of surface roughness and textures at different scales (Johnson and Hsiao, 1994; Bensmaïa and Hollins, 2003). The work by Kappassov et al. (2015) includes a useful summary table in which sensor design criteria are summarized into system characteristics (spatial resolution and number of wires), sensor properties (sensitivity, frequency response, hysteresis) and properties of the outer structural skin layer (hysteresis, surface friction, compliance).

A behavioral approach can be used to characterize artificial fingers from the point of view of their behaviors and functions, more than focusing on the specifications of the sensory system. In particular, metrics have been used to probe behaviorally relevant stimulus quantities analogous to how human perceptual skills are tested (Delhaye et al., 2016). In this case, the well-known sensorised BIOTAC finger is under test. Behaviorally relevant experiments are mainly related to testing the spatio-temporal resolving power of the finger sensing system, and its ability to detect contact stimuli and movement. In particular, experiments are related to localizing contact (localization), measuring the amplitude of the tactile stimuli (pressure discrimination), characterizing motion (motion direction and speed discrimination), and contact surface properties (texture discrimination). The combination of prosthetic fingers and decoders often matches or even outperforms humans under analogous contact stimulation, except for the localization task (due to lower BIOTAC sensor density with respect to that of human mechanoreceptors). The main finding is that the use of relatively few distributed sensors seems not to limit spatial acuity provided that sensors have overlapping receptive fields and biomimetic hyperacuity can be achieved by combining information from adjacent sensors. Regarding the temporal resolving power of the sensing apparatus, at least one high frequency sensor (more than 200 Hz) is needed to measure small vibrations relevant for texture exploration. Nevertheless, it is necessary to test the behavior of this sensing system from dynamic active perspective to fully validate the artificial sensorisation.

A second relevant state-of-the-art study, this time involving a sensing structure which is also producing movements, is reported by Pestell et al. (2018). The authors propose a low-cost 3D-printed optical tactile sensor using a small image tracking chip for high frequency bandwidth and high spatial resolution sensing. Investigated finger capabilities are again focused on the spatial and temporal resolving power of the sensing apparatus. Sensing is the perceptive guidance for two actions. The first is following a given trajectory (e.g., the object contour) whilst maintaining contact with the surface by modulating the contact depth. The second action is precisely identifying a specific position (determined by angle and radial distance) with respect to an edge. The perception of multiple dimensions (depth, angle, distance) is used to successfully guide the robot motion in the closed-loop exploration task of following the contour of a previously unseen object of unknown location. It is worth noting that this is an example of how sensory inputs are transformed and represented as units that are used to drive actions, in line with the concept of affordance specification in driving the processes of action planning and selection in the human brain.

### 3.2. Task-Based Design Approaches

Interpreting haptics at the nexus of perceiving and acting (Prescott et al., 2011) is quite a recent approach in Robotics. Only recently we have witnessed a compelling need to integrate the two spaces. This does not mean that no approaches to that aim have been proposed before, but other challenges seemed more relevant, and because reliable tactile sensors were not available. In passive perception, data acquired by the sensorised fingertip cannot affect the fingertip motion: this approach can be robust if it is used to optimize a specific task, but it is less robust if targeting unstructured environments. On the other hand, in active perception, sensory signals are self-generated and this requires actively moving, seeking information, testing hypotheses. For humans, this happens at the level of a particular haptic feature, but also at a higher level (*What is this object? Where is my door key?*). For robots, this concept of “active” has a local, constrained logic, which is intrinsic to the concept of Artificial Intelligence, at least in its current interpretation. High-level goals are still defined by humans. If the context and high-level goals are defined, in a task-driven approach the robot can be able to manage the task in an optimal way, sometimes even outperforming humans. Dynamically reconfiguring the internal parameters of the system to suit the current task is a well-known concept in Robotics, although typically not conceptually related to active perception. It is noteworthy that not all robot agents for touch are intended for “active touch.” This is something that needs to be carefully engineered, if an active approach is needed.

In the following paragraphs, we concentrate on recent examples of active perception with haptic systems built to optimize a certain task. We use a systematic approach to these use cases and recall keywords that enable an easy mapping to the taxonomy (Figure 3), thus making “behavior,” “goal,” “task,” “sensing,” “sensorimotor architecture,” and “control” explicit. Table 1 supports reading by using the same taxonomy. For robots to operate autonomously in unconstrained environments, active robot perception has been mainly used to *estimate* (for unknown objects) or *classify* (among a set of known objects) the shape of the touched object. Relevant information for shape recognition in humans comes from exploring surfaces, following contours and edges (Germagnoli et al., 1996), and similar approaches have been adopted for robots.

**Table 1:** Haptic behavior, goal, tasks, and sensing system are specified for each selected use case.

**References**	**Behavior**	**Goal**	**Tasks**	**Sensing system**
Matsubara and Shibata, 2017	Exploration of unknown objects	Object shape reconstruction	• Shape estimator • Path planner • Touch point selector	Tactile sensor array (8 FSR sensors)
Martinez-Hernandez et al., 2017	Contour exploration of unknown objects	Object contour extraction	• Adjust local fingertip position until edge orientation can be reliably estimated • Move fingertip tangentially along estimated orientation	iCub tactile sensor array (12 capacitive sensors)
Strub et al., 2014	Dynamic touch (grasping, manipulation, exploration)	Autonomous learning of object shape	• Online adaptation of object shape representation • Online adaption of pose estimate	Tactile sensor array to reconstruct contact pressure distribution
Jamali et al., 2016	Surface exploration of unknown objects	Object shape reconstruction (3D point cloud)	• Fit the best object shape to observed data • Identify next contact location	iCub tactile sensor array (12 capacitive sensors)
Abraham et al., 2017	Ergodic exploration of unknown objects	Object shape estimation	• Collect data by trajectory following • Shape estimation • Define new trajectory to collect highly structured information	Low-resolution binary tactile sensor (contact/no contact)
Sommer and Billard, 2016	Exploration of unknown surface and/or grasping of unknown objects	Creating and maintaining contacts at desired positions on the robot hand	• Maximize the number of contact points • Prevent uneven distribution of contact forces at each contact point	Tekscan tactile sensor array
Bologna et al., 2013	Surface scanning of Braille characters	Online discrimination of Braille inputs	• Scan a given character • Optimize the scanning speed control • Compensate for movement execution errors	Tactile sensor array (24 capacitive sensors)
Sun et al., 2018	Surface exploration	Active strategy for object recognition among a set of previously explored objects	• Explore object along a trajectory at given contact force/velocity • Assess recognition reliability • Set trajectory for next exploration to improve recognition reliability	Commercial proprioceptive (6-axis force-torque) sensor
Martinez-Hernandez et al., 2017	Fast object exploration	Object recognition among a set of previously explored objects	• Data collection: fingers move on the object at given orientation • Choice of the object • If choice is not possible, identify next exploration orientation	Tactile pressure sensor array on fingers plus palm. Proprioceptive sensors (strain plus finger joint angle plus spread motor).

Matsubara and Shibata (2017) propose a method for fast shape estimation (goal) of unknown objects based on active touch-point selection. Active exploration is achieved by implementing a loop including three steps (tasks): shape estimator (representing the object shape given a sequence of touch data); path planner (generating paths from the current robot location to all next touch candidates); touch-point selector (selecting the best point for next touch). The loop ends when the shape estimation converges. The sensorimotor architecture is based on an anthropomorphic arm (7 DoF Barret WAM) endowed with a single finger device, which first collects a touch datum then moves to the next touch-point. Touch-point selection (sensorimotor control) adopts a cost function that suitably combines the uncertainty of the shape estimation and cost of the touch in terms of travel distance. The first term is obtained by modeling a potential function *f*(*x*)∈*R*, where *x* is a point in an n-dimensional Cartesian space. Gaussian Process Implicit Surface (GPIS) models the function *f* given a set of touch data; the uncertainty of the shape estimation is given in analytic forms. The second term stems from a path planner that utilizes the estimated shape to implement the travel cost estimation for all touches to the surface of the (estimated) object; a stochastic model is adopted to tackle this task.

Martinez-Hernandez et al. (2017) present a Bayesian approach for actively controlling a biomimetic fingertip during autonomous exploration (behavior) of an unknown object to extract its contour (goal). Active perception relies on adjusting the local position of the fingertip until the edge orientation can be reliably estimated, and moving the fingertip tangentially along the estimated orientation (tasks). The fingertip is coupled with a Cartesian 2 DoF robot arm spanning *x* and *y* axes, while a Mindstorm NXT Lego robot generates movements in the *z* axis. A tactile exploration based on palpation is chosen. The sensorimotor architecture envisages (i) tapping for 2s over a given location in the *x* − *y* plane while collecting digitized pressure values from the tactile sensor array on the fingertip (sensing); (ii) updating the position of the fingertip if required; (iii) moving the fingertip in the *x* − *y* plane. Bayesian inference supports estimation of edge orientation: position is updated until the degree of belief in the orientation estimation is above a given threshold (sensorimotor control). To support the active repositioning system, 1296 perceptual classes have been generated by tapping against a flat circular object over different locations in the *x* − *y* plane sampled in polar coordinates (data collection). That is, given a fixed angle between sensor and edge, the object is tapped along a line radially extending from the inside of the object (“full contact”), passing over the edge (“partial contact”), and ending outside the object (“no contact”). Accordingly, the 1296 perceptual classes are characterized as many pairs including orientation and position. A “position” aggregates 5 sequential taps in steps of 0.2 mm, thus spanning 1 mm.

In Strub et al. (2014) a neural-dynamic model is proposed for a robot to use dynamic touch (behavior) to autonomously learn the shape of an object (goal). The scheme envisages online adaptation of both object shape representation and pose estimation (tasks). Shunk Dexterous Hand2 is employed with two fingers, each having 2 DoF (i.e., controlled joints). The two phalanges of the fingers are each equipped with tactile sensor arrays to reconstruct the contact pressure distribution (sensing). The robot hand performs an object rotation while recording haptic data, first moving the fingers toward each other until tactile feedback signals sufficient contact with the object, then rotating the object along its *z* axis while controlling the contact force (sensorimotor architecture). The sensorimotor control is based on the following. An object manipulation behavior drives an interactive exploration loop using contact position, orientation, and curvature, respectively, as inputs. Curvature is modeled by the eigenvectors and eigenvalues of the covariance of the pressure distributions, along with the angle of the first eigenvector. All these features are associated with their location in a 3D external coordinate system, computed using forward kinematics. An “estimation path” processes the generated tactile and proprioceptive data to estimate the change of the object pose. A “mapping path” maintains the object model location by extracting allocentric features. A “localizing path” maintains the object pose, i.e., it tracks the object in the 3D external coordinate system. They are both controlled by a “matching module” that uses Dynamic Neural Fields to compare the sensed features with the current object representation in order to decide whether an update of the pose, or the object shape is appropriate.

Jamali et al. (2016) present a method based on active perception that reduces the number of samples required to construct a 3D point cloud of an object to capture its shape (goal), i.e., its edges and surface curvature. In the proposed method, the robot arm with a fingertip endowed with an array of capacitive tactile sensors (sensing) uses a tapping strategy to detect a contact event (when force exceeds a given threshold). At each iteration, collected data are used to construct a probabilistic model of the object surface. The robot selects an *x-y* location, and samples the *z* coordinate, which corresponds to the object surface (sensorimotor architecture). Given a finite number of observed contact locations and associated object heights, the problem is divided in two parts (tasks), i.e., it fits the best object shape to the observed data and identifies the next contact location capturing more information about the object's shape, edges and surface. Both steps are implemented by a supervised learning approach. An iterative process (sensorimotor control) guides surface exploration: a classifier and a regression based on Gaussian Processes are trained by using the sequence of observed contact locations. Then, a new sequence of contact locations is presented as input to the two different predictors. The next contact location is the location where the models have the lowest confidence in their prediction.

High resolution sensing is not always necessary for shape estimation. A low-resolution binary contact sensor (sensing) is utilized to reliably support shape estimation (goal) by ergodic exploration (behavior) in Abraham et al. (2017). Specifically, it is showed that a binary form of tactile sensing (i.e., collision detection) has enough information for shape estimation, when combined with an active exploration algorithm that automatically takes into account regions of shape information. A robot arm carrying a probe in the end-effector has to follow a trajectory modeled as a finite, discrete sequence of locations, and detects a contact, i.e., a sudden change in joint torques (sensorimotor architecture). The sensorimotor control is based on the following sequence: (i) given a trajectory, it collects location and measurement pairs; (ii) it trains a kernel-based classification method that is entitled to approximate the decision boundary between two classes: “collision” and “no collision”; (iii) it transforms the decision boundary (i.e., the shape estimation) into a likelihood distribution; (iv) it uses the estimated posterior distribution to calculate the new trajectory to be completed to search for highly structured information such as shape contours.

Managing shape uncertainty is also useful to optimize object grasping (Li et al., 2016). The uncertainty in object shape is parameterized and included as a constraint in the movement planning stage. The proposed approach is used to plan feasible hand configurations for realizing planned contacts using different robot hand platforms. A compliant finger closing scheme is devised by exploiting both the object shape uncertainty and tactile sensing at fingertips. In another work (Sommer and Billard, 2016) dealing with a robot hand actively complying with unknown surfaces for haptic exploration or grasping (behavior), the goal is to create and maintain contacts at desired positions on the robot hand, while having unilateral constraints on undesired contacts. Tasks maximize the number of contact points while the hand is scanning the surface or grasping the object and preventing an uneven distribution of contact forces at each contact point. The employed dexterous hand (16 DoF AllegroHand with 4 fingers, 4 DoFs each) is controlled using open-loop torques with tactile sensor arrays placed on the inner surface of the phalanxes (sensing). The robot hand has to enclose objects at a predefined position. Using the 7 DoFs Kuka LWR robot, the object is sequentially released and grasped in four other configurations shifted by 2 cm in two different directions, and shifted by 17 deg in two different orientations (sensorimotor architecture). The task execution switches across two modes of sensorimotor control: one controls links not yet in contact, and the other controls the contact force at the joints already in contact. All joints in the fingers that affect force control at the contact points and the desired contact points are controlled in torque. The other finger joints are controlled by a PD controller. At each time step, the control mode for each joint depends on its position relative to the set of contact points C and the set of desired contact points D. The set of joints with existing desired contact points is given by *C* ∪ *D*, i.e., the intersection of current existing contacts and desired contacts; the set of joints with desired contact points is given by *D* \ *C*, i.e., a desired contact but not a contact yet. The mechanism of choosing the desired contact points depends on many criteria, including the robot platform, the task and possible prior on the shape. In the proposed compliant grasping example, all the contact points are considered desired contact points.

In a different perspective, active sensing policies have been used for fine discrimination of Braille inputs (goal) using a closed-loop neurorobotic system (Bologna et al., 2013). The sensing system envisages a fingertip endowed with 24 capacitive sensors responding to mechanical indentation, mounted on a robot arm. Planned tasks are scanning a given character, optimizing the scanning speed control and compensating for movement execution errors. Therefore, the fingertip scans the character at a controlled scanning speed and corrections to the movement trajectory are performed (sensorimotor architecture). Closed-loop sensorimotor control includes (i) two levels of processing implemented by as many levels of spiking networks (ii) a probabilistic classification system for tactile input recognition based on a Naive Bayesian classifier; (iii) a high-level controller that shapes scanning based on optimality classification principles; (iv) a low-level controller for online sensorimotor adaptation. Regarding data collection, 150 trials (scanning) per character have been recorded as training set for the classifier.

At a higher level with respect to extracted features like fine geometrical structures or the whole object shape, there are works related to object reconstruction. This might require a number of steps of feature extraction with features to be combined. How to combine haptic features for recognizing the environment is still not explored in detail. In recent work, Sun et al. (2018) present an object recognition (goal) method that utilizes haptic information gained through surface haptic exploration (behavior). Exploration involves a two-joint finger (with encoders at each joint), with a 6 axis force-torque sensor mounted inside a soft silicone fingertip (sensing). The finger is attached to the UR-3 robot arm, only rotating when necessary to relocate the finger relative to the explored object. The active strategy relies on three tasks, i.e., exploring an object along a trajectory at a given contact force and velocity, assessing the recognition reliability, and setting the trajectory for the next exploration to improve the recognition reliability. The sensorimotor architecture considers first moving the fingertip on the object along a sequence of contact points at given contact (normal) force and velocity: contact locations are obtained by exploiting a contact equilibrium system of equations fed by force-torque measurements. Then, at each contact the pair (normal force, tangential force) is measured. A Bayesian decision rule supports the active strategy for object recognition (sensorimotor control): given all explorations executed at a time *t*, the posterior probabilities for all known objects are computed. If none of the posterior probabilities exceeds a defined threshold, a new exploration is carried out. The training set (data collection) includes 100 different explorations (i.e., trajectories) per object. Accordingly, each object is modeled as a multivariate Gaussian distribution of three features: (i) the average value and (ii) the variance of the friction coefficient over the trajectory, the geometry feature computed by exploiting Iterative Closest Point.

Martinez-Hernandez et al. (2017) propose an approach for object exploration (behavior) and recognition (goal), which allows a robot hand to autonomously inspect interesting object locations to improve perception. The exploration procedure exploits a three-finger robot hand (sensing: 22 tactile pressure sensors on the fingers, 24 on the palm, strain sensors at each finger base), with 1 DoF in each finger to perform opening and closing movements, and 1 DoF for spreading fingers around the palm. It is also possible to acquire proprioceptive information from finger joint angles and from the spread motor in real-time (sensing). A robot arm allows for performing precise exploratory movements in the *x*, *y*, and *z* axes, and rotations in yaw. The sensorimotor architecture for a single exploration envisages that the fingers move to contact the object and stop as soon as a predefined pressure is exceeded, to avoid any damage. Then, the fingers are kept in contact with the object for 1 s, giving enough time to collect 50 samples of position and orientation information from fingers and wrist. For each object to be included in the dataset, a sequence of 30 rotations in yaw and contacts around each object have been performed with rotations of 12 degrees, thereby exploring the complete object (data collection). The active exploration is implemented by a loop involving three steps (tasks and sensorimotor control). The first completes data collection, given an angle orientation as input. The second uses a Bayesian formulation to make a decision on the object (once a belief threshold is exceeded), otherwise the angle orientation is identified for next data collection, by exploiting the information available through the posterior probabilities.

In summary, in all cases above decision making is the most critical step: humans are behind high-level choices which define behaviors, high-level goals and specific tasks. Once these three elements have been defined, together with the context, active touch can be used to find the best strategy to accomplish the specific task using criteria of optimality.

### 3.3. Structure-Based Design Approaches

The way a specific task is accomplished is constrained to the employed platform for touch (e.g., hand, finger), and to its degrees of freedom. In this section, we focus on *dexterous* mechanical structures of the agent for touch with functionalities comparable to human ones (including those with super-human capabilities^2^). These platforms are not restricted to well-defined tasks and could be suitable to open-ended robot behaviors, as discussed in the following section. Among dexterous agents and at the intersection between Neuroscience and Robotics we find those named as *anthropomorphic*. Degrees of freedom here are quite often higher than in common robot hands/fingers and relate to a mechanical structure which tries to mimic the appearance and versatility of a human hand and its grasping mechanisms (Carrozza et al., 2006; Prattichizzo et al., 2013; Mnyusiwalla et al., 2016; Xiong et al., 2016; Zhang et al., 2018).

These approaches are interesting in the context of this paper, including those applications where the human user is involved into the control loop of the artificial hand. Referring to prosthetics, the motivation for this research is that low functionality and controllability together with poor cosmetic appearance are the main reasons why amputees do not regularly use their artificial hands. Concepts like synergies and dimensionality reduction illustrated in the Introduction are of utmost importance in this case. Mimicking humans, possible approaches to simplification consist of coupling some of the hand's degrees of freedom for a reduction of the number of effective inputs. Limiting the number of independent inputs to a few coupled motions has an impact on hand dexterity, improving functions and embodiment of the artificial hand. Postural synergies refer to the coordinated ways humans control dozens of muscles for different hand postures and these principles affect the design of the anthropomorphic hand. A survey of the techniques for dimensionality reduction as well as learning and control strategies built on subspaces of reduced dimension across different fully actuated and underactuated anthropomorphic designs is contained by Ficuciello et al. (2018).

Underactuation involving fewer degrees of actuation than degrees of freedom is the most common approach when designing anthropomorphic hands or fingers. To give a few examples, the biomimetic design of the cybernetic anthropomorphic hand *CyberHand* illustrated in Carrozza et al. (2006) consists of the modular architecture and multilevel control, together with its appearance, kinematics, sensorisation and actuation. A few efferent channels are available, allowing for separate control of each digit as well as thumb finger opposition. As for humans, dimension reduction is also introduced for afferent information, requiring integration from proprioceptive and tactile (exteroceptive) sensors relevant to grasping and holding objects. Interestingly, the control of the artificial hand can be shared between the user and the intelligent mechanics: high-level control interprets the users intention (grasp selection and force level) and low-level control is used to actuate specific grasps and apply the desired total force. In another paper, Xiong et al. (2016) focus on the movement relationship among joints in a digit and among digits in the hand, and on finger postural synergies during grasping. They propose a design theory for the kinematic transmission mechanism to be embedded into the hand palm mimicking postural finger synergies by using a limited number of actuators. Prattichizzo et al. (2013) also develop tools to establish how many task-dependent synergies should be involved in a certain grasp for its stability and efficiency. In the framework of “soft synergies,” they define the correspondence between the controllable internal forces and the motions of the grasped object and the actuated inputs. A recent work illustrates a systematic approach to identify the actuation strategy by studying the correlations of coordinated movements in human hands during 23 grasp tasks (Zarzoura et al., 2019). The authors conclude that 19 degrees of freedom for an anthropomorphic hand can be reduced to 13 degrees of actuation (reduced to 6 by relaxing dimensionality reduction criteria) distributed between six groups of joints. In another recent paper, a continuum mechanism—previously used for manipulator designs—has been applied to an anthropomorphic hand to form synergy-based hand poses in a pre-grasp phase (Xu et al., 2019): three actuators actuate this mechanism to drive eleven hand joints according to two synergy inputs. The designed grasping control strategy presented by Ficuciello (2019) allows an anthropomorphic robot hand to adapt to object contours by means of coordinated finger motion in the synergies subspace, computed through a well-known method for human grasp mapping adapted to underactuated kinematics. Finally, Vulliez et al. (2018) illustrate a novel tendon-driven, bio-inspired design for the fingers of the dexterous RoBioSS hand (4 fingers, 16 DoFs) enabling a relevant simplification of the hand control software with respect to the previous solution characterized by unwanted backlash and nonlinearities (Mnyusiwalla et al., 2016).

## 4. From Task-Based to Structure-Based Designs: The Contribution of Robot Technology and Its Use in Open-Ended Robot Behaviors

While in previous sections we investigated human-centered principles to the definition of robot behaviors, especially in so far as exploration is concerned, here we extend the discussion about how Robotics-related approaches can enable the use of anthropomorphic robot hands in open-ended contexts, as well as what are the main hypotheses involved when robots are used. We analyse two related enabling factors that current research trends in Robotics may bring to the discussion so far. The first posits that the relationships between the most common task-based approach and the formalization of robot behaviors allow for the development of robot behaviors where tasks cannot be clearly defined. The second has to do with the use of general-purpose anthropomorphic robot hands to design and develop open-ended robot behaviors. Such enabling factors include

- The availability of heterogeneous, distributed sensing on the surface of a robot's body, including its hands, along with the representation of tactile information for data processing and control, which is fundamental to integrating human-centered design principles into real robot structures, and
- The need for *computational efficiency* in robot-based sensorimotor loops, which brings about the introduction of bio-inspired solutions.

These two factors are discussed in the paragraphs that follow.

Recent trends in robot-based object manipulation may provide new perspectives for the development of anthropomorphic robot hands inspired by the dexterity of human hands. Such robot hands can leverage the information originating from high-density distributed sensing, and are amenable to use computationally-efficient algorithms to process haptic information for motion planning and control. The computational aspect is of major importance when considering that such devices as next-generation prostheses are meant to be supported by embedded computational systems satisfying both real-time and power-consumption constraints *by design*. While a number of approaches, conceptual methods, and algorithms for interpreting large-scale tactile information have been extensively reviewed by Kappassov et al. (2015) and Luo et al. (2017), such analyses did not address in depth the aspects related to the actual requirements involved in their implementation on resource-constrained, embedded electronic systems.

Since sensory information (although rich and distributed) is useless if it is not employed in effective sensorimotor loops, an effort must be made to deploy techniques to determine a representation (i.e., a map) of the locations where information from sensors originates and to manage integration with other useful sensory modes (e.g., proprioception). The way such representations can ground actual robot perception and control is at the core of the conceptual differences between “geometry-based” and “information-based” representation approaches, as discussed above.

From an historical perspective, and counterintuitively, information-based approaches (including logic-based ones and topographic maps) have been developed before geometry-based approaches, which weight more an abstract view of the robot body. The work discussed by Stiehl et al. (2004), Kuniyoshi et al. (2004), Olsson et al. (2006), Modayil (2010), McGregor et al. (2011), and Noda et al. (2012) exploit logic-based or information theoretic principles to obtain a representation of a robot surface. In these approaches the focus is on the representation *per se*, and as such all aspects related to how the representation can ground actual behaviors at the motor control levels are explored only to a limited extent. A computational model translating contact events in language-like discrete symbols with well-defined semantics has been presented by Stiehl et al. (2004). Tactile features are rendered as logic symbols, to be used in action planning, with the aim of obtaining a high-level, cognitive representation out of tactile data. There is no direct connection with motion control, action planning being the reference level which the representation interacts with. We observe that the presence of logic symbols associated with certain tactile features would be a nice-to-have feature at the decision making level in robots, where context-based information can be used to decide which task or behavior is more relevant or urgent, irrespective of the involved motor actions. The work presented by Kuniyoshi et al. (2004) faces the problem of learning topographic representations by physically interacting with the environment. Via simulations, it is demonstrated how it is possible to build a tactile body map by temporally correlating signals from tactile sensors. Such maps give priority to the detection of selected, more frequent, patterns of activity, which is fundamental to attain reactive robot behavior. While this approach does not yield novel insights into the problem of translating a rich and complex representation into a motion control problem of lower dimensionality, it provides interesting hints about how information could bootstrap autonomous behaviors in robots or next-generation prosthetic limbs. A task-based representation of a robot body and its functions is generated, together with an optimal sensory flow, which is expected to optimize such tasks.

Along these lines, frameworks using similar principles have been presented in Olsson et al. (2006), Modayil (2010), McGregor et al. (2011), and Noda et al. (2012). The work described in Olsson et al. (2006) and McGregor et al. (2011) is aimed at building sensoritopic maps of groups of sensors using self-organizing processes. This approach produces a representation describing the logical correlations among sensors, but it does not quantitatively represent where, on the robot surface, they are. Sensoritopic maps maintain a logic structure where points of high correlations (in terms of synchronous sensor activations) become very close in the map (on a statistical basis), where spatially and temporally uncorrelated sensors are far from each other. It is evident how such a representation cannot be directly mapped to robot perception or control processes. It defines a data processing information flow playing a role when huge amounts of sensory data must be processed in close to real-time. A similar approach is pursued by Modayil (2010). Feedback from groups of sensors has been used by Noda et al. (2012) to determine somatotopic connections between spatially and temporally correlated elements. All in all, information-based approaches can effectively *build* a representation of the sensors distributed on a large-scale surface, but they cannot be easily employed to implement such functions as autonomous manipulation tasks, since their integration with motion control is difficult to implement. In the Robotics literature, it has been argued that the missing link between the representation of sensory information and robot motion control is a well-defined geometrical characterization of the robot's surface, and therefore such a characterization has been sought by Cannata et al. (2010b), Cannata et al. (2010a), Del Prete et al. (2011), Mittendorfer and Cheng (2012), and Denei et al. (2015). Based on the preliminary work presented by Cannata et al. (2010b), surface parameterization techniques have been employed to obtain a 2D topographic map of a robot surface provided with tactile sensors (Cannata et al., 2010a; Denei et al., 2015). Such a map is able to uniquely represent different parts of the surface in the same metric space, therefore paving the way for general-purpose data-processing algorithms. The work discussed by Mittendorfer and Cheng (2012) is able to retrieve the position of tactile elements in 3D space using *a priori* knowledge about both robot shape and inertial information. Such an architecture could enable autonomous, pose-dependent robot hand behaviors by integrating multi-modal (tactile and inertial) information, thereby allowing for dedicated autonomous hand motions, e.g., dexterous grasping, in response to specific tactile events or motion patterns.

Once a representation of the tactile space is available, the need arises to correlate it with perception processes and sensorimotor loops to implement actual autonomous behavior. As far as the scope of this paper is concerned, the work in Stober et al. (2011) is worth discussing. It shows how sensorimotor loops and robot-environment interaction allow for the creation of structure and correlations in sensory information. It is discussed therein how a robot is able to acquire an egocentric body model including sensory geometry and structure, and how such sensorimotor structure can be used by high-level, autonomous robot behavior. These facts are particularly relevant to enable such functions as *tactile servoing* or *slip detection* during contact regimes subject to gravity and other external forces, since they allow for mapping patterns of tactile data with autonomous behaviors not mediated by high-level reasoning processes, as it would be required for reactive behaviors in prosthetic devices. It is noteworthy that, as far as robot grasping is concerned, such mappings have been developed by De Souza et al. (2015) to infer grasp intentions in humans and by El-Khoury et al. (2015) to synthetise robot grasping behaviors for the specific task at hand.

## 5. Discussion and Conclusions

**Figure 5:**
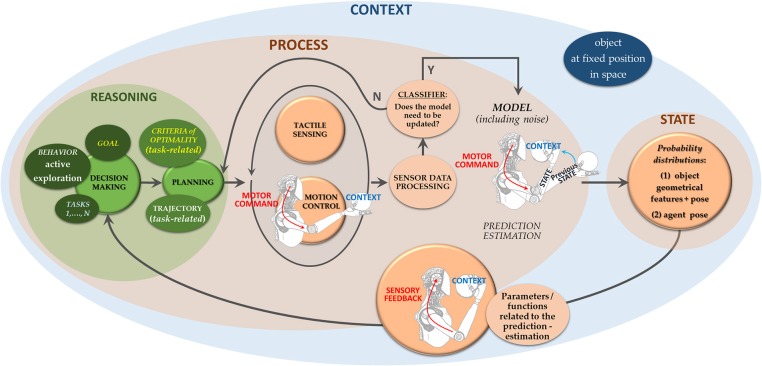
Taxonomy for active exploration based on probabilistic approaches: the scheme reported in Figure 1 has been enriched with conceptual pictures adapted from human motor control theory (Wolpert and Ghahramani, 2000) into robot-like schemes (with permission from the authors of the original pictures). In particular, at each step of the sensorimotor loop a model of the system state is done, which relies on noisy data. A representation is thus given of the system *state* which is based on probability distributions of features and poses. Related parameters and functions are fed back to the reasoning stage to plan and control the next motion step. Sensor data acquired during motion enables the system to tune the model according to the new sensory information.

Today's new-generation robots are increasingly equipped with more sensing components and consequently they are (to some extent) able to deal with highly complex and dynamic real-world tasks. Human-like perceptual qualities have been discussed by Delhaye et al. (2016) and Pestell et al. (2018) involving artificial fingers based on distributed heterogeneous sensors. The proposed approaches demonstrate that such dynamic sensing qualities of artificial fingers are particularly relevant for active explorative behaviors. While completely handling open-ended active behaviors is not mature as a feature yet because, conceptually, Artificial Intelligence is bound to the concept of a closed-world model, first attempts toward specific active approaches involving well-defined tasks can be found in the recent literature pertaining to the niche of Cognitive Robotics. Robust active exploration remains a challenging problem, and major practical hurdle in the deployment of exteroceptive and proprioceptive sensors. It is worth noting that this approach is not the gold standard; in contrast, active exploration is still cutting-edge and experimental in Robotics research, and underappreciated. Some attempts to organize movements based on autonomous robot ability with fingers acting as effective information-seeking agents can be found in task-based approaches (section 3.2). The work by Strub et al. (2014) is an interesting example of how a representation of an object shape can be autonomously built by a robot through dynamic touch, a single term used in the literature on human haptic perception to include different robotic behaviors. All use-cases discussed in this paper can be summarized within the general scheme proposed in section 2. To enable the reader to navigate that section and the whole paper through pictures, we recall that Figure 1 illustrates the proposed scheme for closed loop sensorimotor control of robot agents for touch, Figure 2 depicts the transition from sensor data to system representation, Figure 3 details all sub-blocks and categories of Figure 1, and finally Figure 4 concludes with how specific exploratory procedures are linked to corresponding haptic features. Not surprisingly, movability is not yet a keyword in the Robotics literature on active touch, as objects are often quite-artificially constrained to fixed positions during active exploration so as not to add a degree of complexity to the challenging problem of shape estimation or classification. For those use-cases based on probabilistic approaches, we include in our scheme a useful framework from leading human motor control theory (Wolpert and Ghahramani, 2000), as illustrated in Figure 5. This helps in showing how a theoretical framework developed from human movement science has grounded and inspired robot control solutions within the context of well-defined tasks, which in turn reflects preliminary steps toward autonomous robot exploration.

Beyond the current state of the art, human-inspired principles and current research in Robotics provide valuable insights for the advancement of future *general-purpose* sensorimotor systems for robots. Novel platforms based on anthropomorphic mechanics of the artificial hand are now available, which might be suitable to handle more open-ended tasks in the future, in that they mimic the potentialities of the human hand which is the result of thousands of years of human evolution. Robots may use prior experience to learn about new objects they have not encountered. To give a recent example, zero-shot learning has been applied to mediate haptic recognition of new objects using an anthropomorphic robot hand (Abderrahmane et al., 2018). However, the objective of having autonomous robots successfully manage a variety of tasks in uncertain environments is yet far from realization. As stated in the Introduction, the remarkable degree of functional flexibility of the human hand across extensive variations in conditions is the result of both extraordinary anatomical design and highly complex underpinning central nervous system control processes. The use of anthropomorphic robot hands suitable to operate in open-ended contexts, not constrained to well-defined tasks, may benefit from the recent availability of large-scale, distributed tactile sensing in Robotics, enabling human-like perceptual capabilities. Moreover, the absence of constraints on the types of hand motions and possible hand-object interactions is likely to require the detection and interpretation of heterogeneous contact phenomena spanning the whole hand surface. Obviously, this requires the deployment of an increased ratio between the number of sensors and the surface of interest, for instance, a huge number of tactile sensors on a small robot fingertip, as required on an anthropomorphic robot arm or next-generation prosthetic limb. Managing these complex systems will require implementing sensorimotor control mechanisms that draw from such concepts as sensor binding, representation, dimensionality reduction and motor synergies. In particular, an effort must be made to design, develop and deploy techniques to determine a representation of sensor locations, and the relationships between the tactile domain and other useful sensory modes, such as proprioceptive sensing. This is where some current research trends in Robotics might bring an important contribution.

Much of the current research in Robotics about the exploitation of large-scale tactile information is aimed at addressing two related challenges. On the one hand, the representation and management of heterogeneous, distributed sensory information originating from large-scale robot surfaces; on the other hand, the adoption of a real-time computational infrastructure to collect and process sensory data for motion planning and control. Both of these challenges are strongly related to the geometry-based and information-based paradigms introduced above, as well as to the task-based or structure-based design approaches, discussed in this paper. Analysing these two challenges in light of these two dichotomies, a few novel principles for the design of human-like robot hands that are apt to work in open-ended contexts can be proposed, for example as far as autonomous grasp control and active exploration (and, in the future, dexterous manipulation) are concerned.

In any case, the location of tactile elements with respect to one or more robot-centered reference frames (in a full analogy with what has been hypothesized for humans), and the correspondence between those tactile elements and their representation for control, must be determined without ambiguity. As a consequence, any future control framework for human-like, anthropomorphic robot hands that includes sensorimotor loops based on tactile information for perception and motion control will need to address the following specific questions:

- How can a “useful” (i.e., for perception and control aspects), computationally efficient, representation of an artificial hand surface (and its sensors) be automatically generated from tactile elements, which is able to guide autonomous, open-ended behaviors for which a robot has not been designed to solve in advance?
- Is it possible to obtain this kind of representation allowing for the design of tactile data-processing algorithms independent of the peculiar shape and morphology of the robot hand, and enable adaptive solutions to open-ended tasks?
- How to exploit such a representation for goal-oriented tactile-based robot behaviors?

A number of approaches in the past few years have addressed these questions in Robotics, and specifically the representation problem, proposing solutions and models at different levels of abstraction, encompassing geometry-based representations, logic-based principles, and topographic maps. Needless to say, the specific approach to the way an anthropomorphic hand structure as well as its sensors are represented has a strong impact on the kind of sensorimotor loops (including all the haptic processes) that can be grounded.

Information-based approaches are appealing for enabling autonomous behaviors in human-like, anthropomorphic robot hands by offering plausible mechanisms for building and maintaining knowledge of the relationships between specific contact events and how they are processed for control purposes. In other terms, information-based approaches may represent a key means to allow for symbol grounding as posited by Harnad (1990). However, dynamic adaptation mechanisms are necessary for an anthropomorphic robot hand to learn new somatotopic correlations as new tasks are carried out, i.e., a high-level of acute plasticity in the design of perception, representation and control components is required for a truly flexible and adaptive robot hand. Moreover, information-based approaches cannot be easily employed to implement autonomous behaviors, since their integration with motion control is difficult to implement.

While in information-based approaches representation criteria may not be related to Euclidean metrics, geometry-based representational approaches can help in managing data coming from heterogeneous sensors distributed over complex 3D structures. Being able to abstract shape from a given surface is of the utmost importance for distributed, modular and scalable data processing architectures, since this enables the distribution of computational units in the mechanical structure of the artificial hand device irrespective of its mechanical design, thus addressing the second question posed above.

To conclude, to make adaptive, goal-directed “choices,” the problem of how to implement high-level goals remains. A bottleneck here is still the decision-making step for high-level goals (in any case, whether humans should strictly govern the control of robots or not is certainly among the most contentious ethical issues related to active robot control). This might be partially solved when anthropomorphic-based approaches guide the design of artificial limbs for prosthetics, in that the human brain is directly integrated into the control loop and might be the agent responsible for high-level choices. Depending on the requirements for the task at hand, however, some intelligence embedded into the prosthesis can be envisaged. First attempts in this direction are related to the prosthesis' automatic control of incipient slip, e.g., Zhang et al. (2018).

In the future, other forms of advanced haptic intelligence may be embedded into prostheses, and human psychology and neuroscience may provide inspiration for this. As noted in the Introduction, there is evidence for modular task-dependent solutions in the functional organization of the human brain. Object recognition and the control of actions are governed, at least in part, by anatomically and functionally separable brain systems, —the same haptic information is used, yet organized differently according to the task. These concepts might be applied to the management of haptic feedback in prosthetics. Task-dependent processing of haptic information might be mimicked by an Artificial Intelligence embedded in the prosthetic device: sensor redundancy might be used to extract rich haptic information from heterogeneous sensor data and the interface with the human can be—either by the human or by the prosthesis itself—adaptively configured to send back to the user different haptic information depending on the task.

Completely new scenarios are also envisaged in which multisensory integration can be used together with acutely plastic brain mechanisms to enable dynamic user-device solutions that transform over time. Basic neuroscience questions may also arise from such advancements, such as what are the necessary conditions for the brain to build feedforward models of the prosthetic limb, and what neural changes accompany learning to use a prosthetic device where visual and tactile signals caused by a single event (e.g., one object touching the skin) are physically displaced and delayed in time.

In summary, there is no shortage of possible contributions that research in Robotics can deliver to the design of novel human-like robotic hands, especially where the transition from task-based to structure-based design is concerned. These contributions rely on continuing advancements that are both cognitive and physical in nature, such as efficient data representation, real-time processing and embedded networking. However, it is apparent that these feature advancements cannot be considered in isolation, since a number of interdependences affect various levels of design, as this brief discussion suggests, one of these dependences being the relationships entailed by the robot structure and the environment where it operates (Marcel et al., 2017).

## Author Contributions

LS: main conceptualization and definition of the overall narrative structure, literature investigation, picture design and realization, and paper writing. PG: literature analysis, conceptualization of task-based approaches, and main contributor to section 3.2. SW and KV: conceptualization, introduction writing, review, and editing of the whole manuscript. FZ: literature database. FM: supervision, main conceptualization and definition of the overall narrative structure, main contributor of section 4, contributions to discussion/conclusion, review, and editing of the whole manuscript.

### Conflict of Interest Statement

The authors declare that the research was conducted in the absence of any commercial or financial relationships that could be construed as a potential conflict of interest.

## Footnotes

^1^It is worth noting that the term “reasoning” has been used differently by other authors, associated with the step before decision making (Bajcsy, 1988).

^2^It is still unknown whether better hands for manipulation than the human hand exist or not, and the relationship between the manipulative capability and hand morphology needs to be investigated in the region of more possible hand morphologies (Hu et al., 2018).
